# Association between the systemic inflammatory response index and mortality in patients with sarcopenia

**DOI:** 10.1371/journal.pone.0312383

**Published:** 2024-11-18

**Authors:** Yifan Lu, Chengyin Lu, Zhiqiang Luo, Pei Chen, Hui Xiong, Wangyang Li

**Affiliations:** 1 The Second Hospital of Hunan University of Chinese Medicine, Changsha, Hunan, China; 2 Hunan University of Chinese Medicine, Changsha, Hunan, China; 3 The First Hospital of Hunan University Chinese Medicine, Changsha, Hunan, China; Hacettepe University Cancer Institute, TÜRKIYE

## Abstract

**Background:**

Sarcopenia is closely linked to inflammation; however, the association between the systemic inflammatory response index (SIRI) and mortality in patients with sarcopenia remains unclear. This study aims to explore the relationship between SIRI and mortality in sarcopenia patients.

**Methods:**

We analyzed data from ten cycles of the National Health and Nutrition Examination Survey (NHANES) spanning 1999 to 2018, selecting 3,141 sarcopenia patients. Mortality data were obtained from the National Death Index up to December 31, 2019. Participants were divided into three groups based on the ranking of their SIRI values. The association between SIRI and mortality was assessed using Cox proportional hazards models, with smooth curve fitting employed to test the correlation. Sensitivity analyses, subgroup analyses, and interaction tests were conducted to validate the stability of the findings.

**Results:**

A total of 101,316 individuals were included in this study. During a median follow-up of 10.4 years (minimum follow-up time of approximately 0.08 years, maximum follow-up time of 20.75 years), 667 participants died. Kaplan-Meier (KM) analysis indicated a higher risk of mortality in the SIRI Q3 group. Cox regression analysis showed a significant association between the SIRI Q3 group and all-cause mortality [HR 1.24 (95% CI: 1.05, 1.47)] and cardiovascular disease mortality [HR 1.46 (95% CI: 1.04, 2.04)]. Subgroup analysis revealed that SIRI was significantly associated with all-cause mortality across various demographic characteristics (e.g., gender, diabetes, hypertension, cardiovascular disease). Sensitivity analysis, excluding participants with cardiovascular disease, those who died within two years of follow-up, and those under 50 years old, indicated higher hazard ratios (HRs) for all-cause and cardiovascular mortality in the SIRI Q3 group.

**Conclusion:**

This study demonstrates a significant association between SIRI and an increased risk of mortality in sarcopenia patients aged 20 years and older.

## 1. Introduction

As society gradually ages, sarcopenia has emerged as an increasingly serious health issue. This condition is characterized by a marked decline in skeletal muscle mass and strength, significantly impairing mobility and quality of life in the elderly [1]. The progressive weakening of muscles makes walking difficult, leading to a heightened risk of falls and fractures [2]. Furthermore, the deterioration of physical function forces individuals to become dependent on others, which increases psychological burdens and may lead to mental health issues such as depression. At the same time, patients with sarcopenia require more medical services, including rehabilitation and inpatient care, which places an additional economic burden on families and society. The cost of treating and managing sarcopenia and its complications is substantial, imposing a heavy financial strain on public health systems. With the aging population trend intensifying, the prevalence of sarcopenia continues to rise. It is noteworthy that sarcopenia not only affects the elderly but can also cause pre-retirement individuals to leave the workforce prematurely due to declining physical function, thereby affecting overall societal productivity. Therefore, the prevention and treatment of sarcopenia are of critical importance. Additionally, sarcopenia is closely linked with other conditions such as osteoporosis, metabolic disorders, cardiovascular diseases, and malnutrition [3–7].

Research indicates that the inflammatory response plays a crucial role in the development of sarcopenia, with elevated levels of inflammation impairing muscle mass and affecting gait in older adults [8, 9]. The systemic inflammatory response index (SIRI), an index based on the ratio of neutrophils, monocytes, and lymphocytes, is widely used to assess the prognosis of gastric cancer, colorectal cancer, and cardiovascular diseases, effectively evaluating the immune and inflammatory status of the body [10–13].

Although current studies suggest that inflammation markers are elevated in patients with sarcopenia, the relationship between SIRI and mortality in these patients has not been fully elucidated. Therefore, this study aims to explore the association between SIRI and both all-cause and cause-specific mortality in sarcopenia patients. By clarifying this relationship, the study underscores the necessity for innovative therapeutic approaches and proposes directions for future research, thereby offering new perspectives for the management and prevention of sarcopenia.

## 2. Methods

### 2.1 Study population

**Fig 1:**
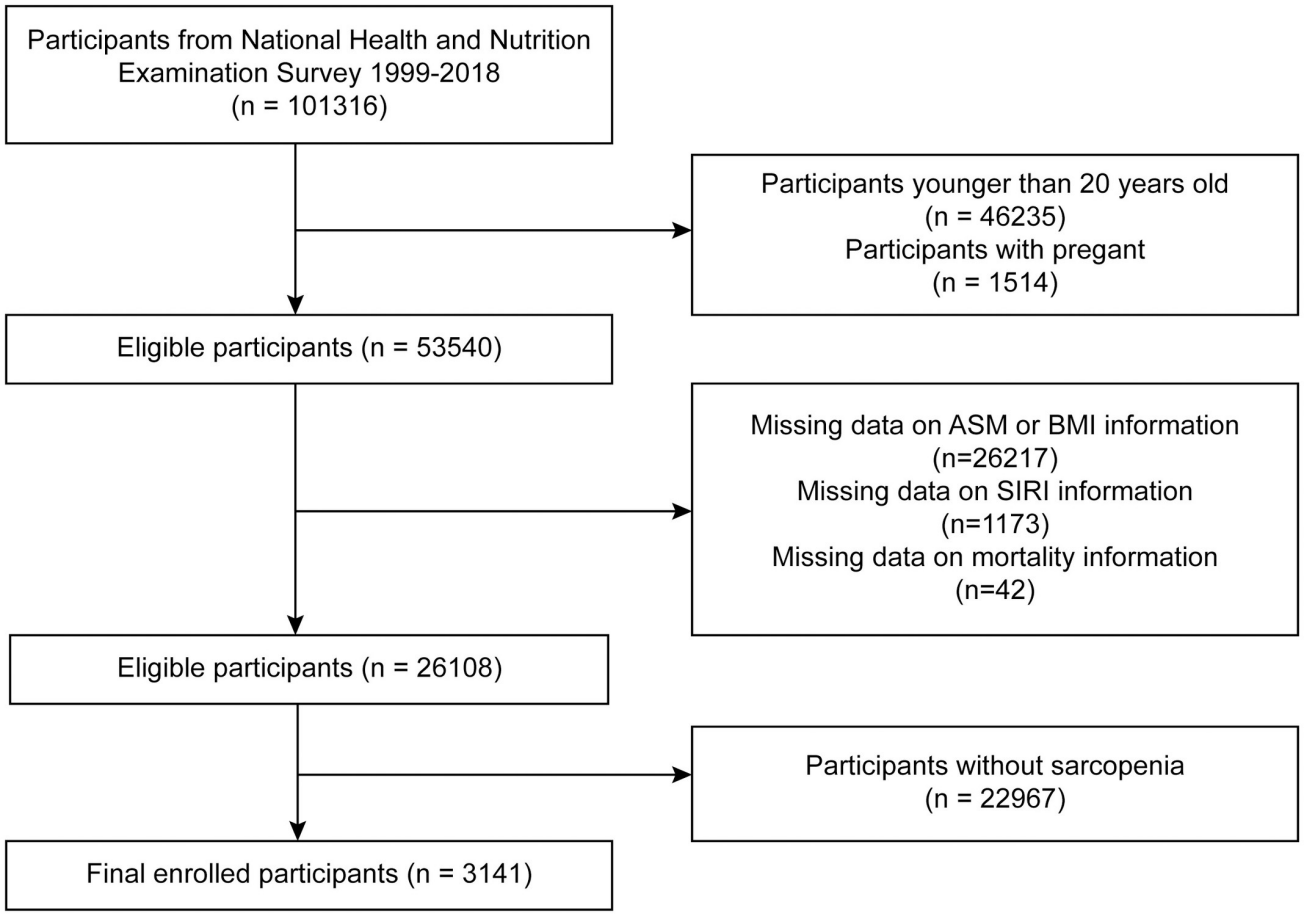
Participant screening flowchart.

The National Health and Nutrition Examination Survey (NHANES) is a nationwide survey conducted by the Centers for Disease Control and Prevention (CDC) aimed at assessing the health and nutritional status of the U.S. population. Each NHANES cycle spans two years, during which approximately 5,000 U.S. residents are selected annually for detailed interviews and physical examinations using mobile examination centers. The survey collects a wide range of health-related data, including demographic information, socioeconomic status, health status, medical history, dental health, physiological measurements, and laboratory test results. All data used in this study are publicly available on the CDC website (www.cdc.gov/nchs/nhanes). This study was reviewed and approved by the National Center for Health Statistics (NCHS) of the CDC. Written informed consent was obtained from all participants in the study. The sample for this study was drawn from eligible participants in all NHANES cycles conducted between 1999 and 2018, totaling 101,316 individuals. Exclusion criteria included individuals under 20 years of age (n = 46,235), pregnant women (n = 1,541), participants missing data on sarcopenia (n = 26,217), those missing SIRI data (n = 1,173), those without mortality data (n = 42), and those who did not have sarcopenia (n = 22,967). Ultimately, our analysis included 3,141 participants with sarcopenia who had complete SIRI and mortality data. The detailed screening process is illustrated in Fig 1.

### 2.2 Definition of research variables

The Systemic Inflammatory Response Index (SIRI) is a measure used to assess systemic inflammatory status, defined as the product of monocyte and neutrophil counts divided by the lymphocyte count. In this study, sarcopenia was defined according to the criteria established by the European Working Group on Sarcopenia in Older People (EWGSOP) and the Asian Working Group for Sarcopenia (AWGS) [14, 15]. Specifically, sarcopenia was identified based on the ratio of Appendicular Skeletal Muscle (ASM) to Body Mass Index (BMI), with threshold values of ≤ 0.789 for men and ≤ 0.512 for women. ASM, which refers to the lean muscle mass of the appendages (upper and lower limbs) excluding bone mineral content, was used as a key measure. Participants with an ASM/BMI ratio below these thresholds were classified as having sarcopenia.

Covariates for this study were derived from the NHANES database and included: (1) Demographic variables such as gender, age, race/ethnicity, education level, marital status, and poverty income ratio (PIR). (2) Diabetes (DM), hypertension, hyperlipidemia, and cardiovascular disease (CVD) were defined based on physical examination results, laboratory data, questionnaire responses, and standards from relevant associations. Laboratory data included serum alanine aminotransferase (ALT), aspartate aminotransferase (AST), and urine albumin-to-creatinine ratio (UACR), which were the only biochemical markers extracted from the NHANES database. (3) Behavioral characteristics such as smoking (never, former, current) and alcohol use (never, former, light, moderate, and heavy).

### 2.3 Mortality data

Mortality data for this study were obtained from the NHANES database, with a cutoff date of December 31, 2019. Follow-up time was calculated in months, starting from the date participants were enrolled in the NHANES program at a mobile examination center, and continued until the date of death or the end of the follow-up period. The primary mortality outcomes of interest in this study were all-cause mortality, cardiovascular disease mortality, cancer mortality, and chronic lower respiratory disease mortality.

### 2.4 Statistical analysis

Due to the lack of a standardized threshold for normal SIRI values, participants in this study were divided into three groups based on SIRI tertiles. Missing data for covariates were handled using the random forest imputation method. Continuous variables are expressed as Mean ± SD, while categorical variables are shown as percentages.

Cox proportional hazards regression analysis was used to examine the relationship between SIRI and all-cause mortality in sarcopenia patients. Three different models were established: Model 1 included only SIRI without adjusting for any confounders; Model 2 further adjusted for age, gender, and race; and Model 3 additionally included education level, PIR, marital status, as well as ALT, AST, UACR, DM, hypertension, hyperlipidemia, CVD, alcohol use, and smoking. The same modeling framework was applied to analyze cause-specific mortality. Additionally, the log hazard ratio (log HR) of SIRI and its relationship with mortality were assessed using smooth curve fitting plots. Interaction tests and stratified analyses were conducted to evaluate potential effect modifications by factors such as age (using 60 years as the threshold), gender, DM, CVD, race, marital status, education, and hyperlipidemia.

Three types of sensitivity analyses were conducted to verify the robustness of the association between SIRI and mortality in sarcopenia patients. First, all patients diagnosed with cardiovascular disease at baseline were excluded to assess the independent predictive ability of SIRI in individuals without CVD. Second, to minimize the potential impact of early mortality on the results, participants who died within two years of follow-up were excluded. Third, considering the impact of age on sarcopenia and mortality, all participants under 50 years old were excluded to evaluate the predictive ability of SIRI in older populations.

Furthermore, to explore the role of SIRI across different groups, all participants, including those without sarcopenia, were re-categorized. Participants were divided into high and low SIRI groups, which were then combined with the presence or absence of sarcopenia to form four subgroups. This allowed for further investigation of the interactive effects of SIRI and sarcopenia on mortality. Package R and Empower Stats were used for statistical analysis, with *p* < 0.05 considered statistically significant.

## 3. Results

### 3.1 Baseline characteristics

This study involved the analysis of data from ten NHANES cycles, resulting in a total of 3,141 participants, as illustrated in Fig 1. The demographic characteristics and laboratory data of the participants are summarized in Table 1, and they were divided into tertiles based on their SIRI values: the first tertile (Q1, n = 1047; SIRI ≤ 0.912), the second tertile (Q2, n = 1046; 0.912 < SIRI ≤ 1.464), and the third tertile (Q3, n = 1048; SIRI > 1.464). The average age of these participants was 56.1 years, with males comprising 51.51% of the cohort. The mean SIRI value was 1.4 with a standard error of 0.018. The Q3 group was characterized by an older age, a higher proportion of males, and predominantly non-Hispanic Whites. Higher education levels and a greater proportion of unmarried individuals were also more prevalent in the Q3 group. The proportions of smoking and alcohol use were lower in the Q3 group, and the prevalence of diabetes mellitus (DM), hypertension, and cardiovascular disease (CVD) increased significantly with higher SIRI values.

**Table 1:** Characteristics of the study population based on systemic inflammatory response index (SIRI) tertiles.

Variables	Q1(n = 1047)	Q2(n = 1046)	Q3(n = 1048)	*P* value
AGE	53.32 ± 15.69	55.85 ± 17.39	59.16 ± 18.30	<0.001
ALT	28.73 ± 18.60	27.69 ± 20.54	27.49 ± 20.54	0.309
AST	26.60 ± 12.67	25.67 ± 12.59	25.68 ± 14.25	0.178
UACR	63.53 ± 601.46	86.02 ± 544.85	82.47 ± 304.02	0.543
SIRI	0.67 ± 0.16	1.17 ± 0.16	2.37 ± 1.21	<0.001
GENDER				<0.001
Male	429 (40.97%)	534 (51.05%)	655 (62.50%)	
Female	618 (59.03%)	512 (48.95%)	393 (37.50%)	
RACE				<0.001
Mexican American	515 (49.19%)	445 (42.54%)	323 (30.82%)	
Other Hispanic	90 (8.60%)	95 (9.08%)	79 (7.54%)	
Non-Hispanic White	269 (25.69%)	402 (38.43%)	538 (51.34%)	
Non-Hispanic Black	67 (6.40%)	31 (2.96%)	53 (5.06%)	
Other Race Including Multi-Racial	106 (10.12%)	73 (6.98%)	55 (5.25%)	
EDUCATION				<0.001
Less Than 9th Grade	313 (29.89%)	335 (32.03%)	243 (23.19%)	
9-11th Grade (Includes 12th grade with no diploma)	188 (17.96%)	161 (15.39%)	178 (16.98%)	
High School Grad/GED or Equivalent	236 (22.54%)	221 (21.13%)	256 (24.43%)	
Some College or AA degree	195 (18.62%)	225 (21.51%)	238 (22.71%)	
College Graduate or above	115 (10.98%)	104 (9.94%)	133 (12.69%)	
MARITAL STATUS				0.094
Married	625 (59.69%)	631 (60.33%)	588 (56.11%)	
Living with partner	60 (5.73%)	59 (5.64%)	46 (4.39%)	
Never married	115 (10.98%)	100 (9.56%)	131 (12.50%)	
Other	247 (23.59%)	256 (24.47%)	283 (27.00%)	
PIR				0.651
High	195 (18.62%)	190 (18.16%)	201 (19.18%)	
Medium	451 (43.08%)	466 (44.55%)	478 (45.61%)	
Low	401 (38.30%)	390 (37.28%)	369 (35.21%)	
SMOKING				<0.001
Never	648 (61.89%)	585 (55.93%)	481 (45.90%)	
Former	272 (25.98%)	297 (28.39%)	355 (33.87%)	
Now	127 (12.13%)	164 (15.68%)	212 (20.23%)	
ALCOHOL USE				<0.001
Never	238 (22.73%)	201 (19.22%)	148 (14.12%)	
Former	169 (16.14%)	167 (15.97%)	166 (15.84%)	
Mild	337 (32.19%)	391 (37.38%)	434 (41.41%)	
Moderate	133 (12.70%)	111 (10.61%)	134 (12.79%)	
Heavy	170 (16.24%)	176 (16.83%)	166 (15.84%)	
DIABETES				0.004
No	811 (77.46%)	781 (74.67%)	745 (71.09%)	
Yes	236 (22.54%)	265 (25.33%)	303 (28.91%)	
HYPERLIPIDEMIA				0.114
No	728 (69.53%)	737 (70.46%)	770 (73.47%)	
Yes	319 (30.47%)	309 (29.54%)	278 (26.53%)	
HYPERTENSION				<0.001
No	682 (65.14%)	583 (55.74%)	498 (47.52%)	
Yes	365 (34.86%)	463 (44.26%)	550 (52.48%)	
PRECVD				<0.001
No	926 (88.44%)	882 (84.32%)	787 (75.10%)	
Yes	121 (11.56%)	164 (15.68%)	261 (24.90%)	

Mean ± SD for continuous variables: *P* value was calculated by linear regression model.

% for categorical variables: *P* value was calculated by chi-square test.

### 3.2 Association of SIRI with all-cause and cause-specific mortality

**Fig 2:**
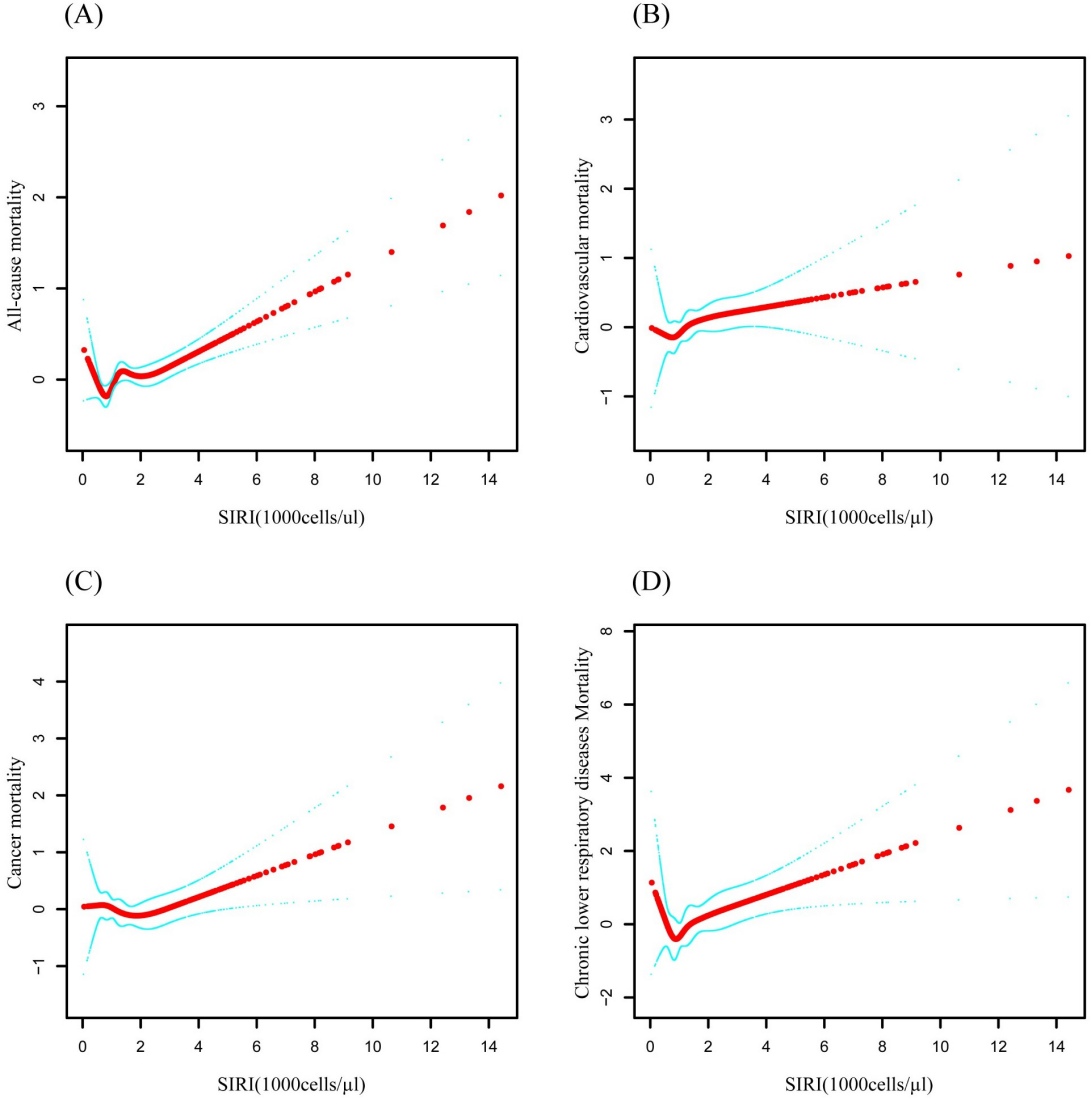
Smooth curve-fitting relationship between systemic inflammatory response index (SIRI) and mortality rate.

**Fig 3:**
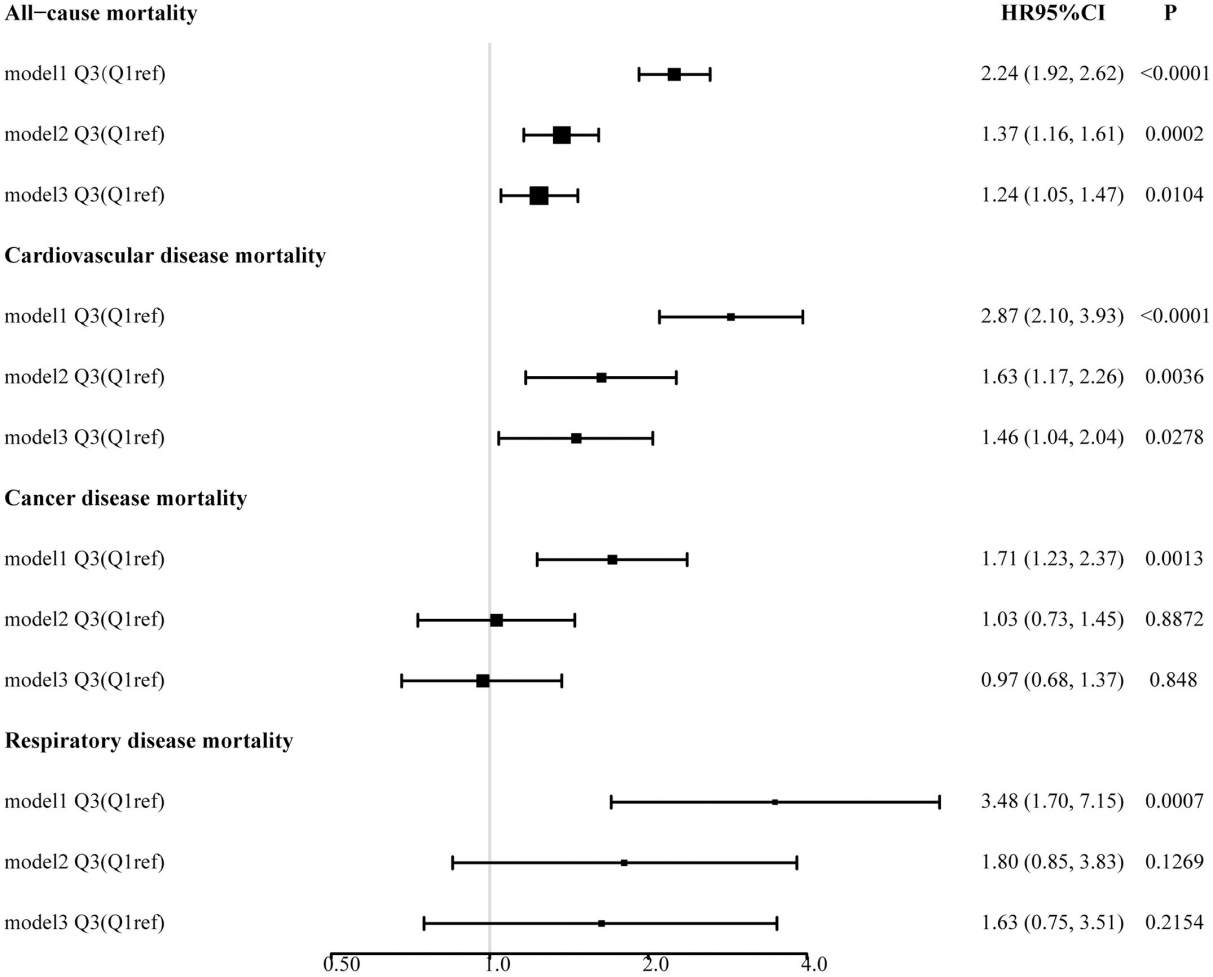
Forest plot of the relationship between systemic inflammatory response index (SIRI) and mortality from sarcopenia.

**Fig 4:**
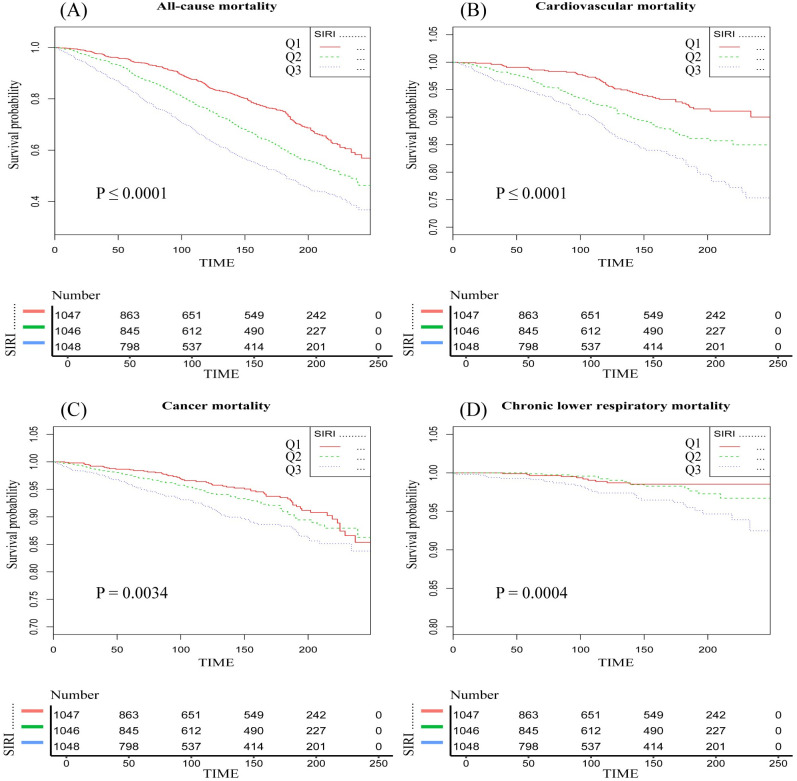
Kaplan-Meier curves of the relationship between systemic inflammatory response index (SIRI) and mortality in sarcopenia.

During the median follow-up period of 10.4 years (with a maximum of 20.75 years), a total of 667 deaths occurred. Given the lack of a universally accepted SIRI threshold, we first applied a smooth curve fitting method, which revealed significant nonlinear relationships between higher SIRI levels and all-cause mortality, cardiovascular disease mortality, cancer mortality, and chronic lower respiratory disease mortality (Fig 2). In the COX analysis, Model 1 for the Q3 group showed associations with all-cause mortality [HR 2.24 (95% CI: 1.92–2.62)], cardiovascular disease mortality [HR 2.87 (95% CI: 2.10–3.93)], cancer mortality [HR 1.71 (95% CI: 1.23–2.37)], and chronic lower respiratory disease mortality [HR 3.48 (95% CI: 1.70–7.15)]. In Model 3, the Q3 group showed associations with all-cause mortality [HR 1.24 (95% CI: 1.05–1.47)], cardiovascular disease mortality [HR 1.46 (95% CI: 1.04–2.04)], cancer mortality [HR 0.97 (95% CI: 0.68–1.37)], and chronic lower respiratory disease mortality [HR 1.63 (95% CI: 0.75–3.51)] (S1 Table, Fig 3). To ensure the reliability of the results, we calculated trend *p*-values for SIRI levels across different models, which ranged from 0.304 to 0.370, supporting the robustness of the nonlinear relationships. Kaplan-Meier (KM) analysis indicated significant differences in mortality across different types, with *p*-values ranging from 0.0034 to <0.0001 (Fig 4).

### 3.3 Subgroup analyses

Table 2. Subgroup analyses revealed that SIRI was significantly associated with all-cause mortality in the following subgroups: participants aged 20–60 years [HR 1.87 (95% CI: 1.21–2.90)] and 61–85 years [HR 1.72 (95% CI: 1.45–2.05)]; males [HR 1.40 (95% CI: 1.12–1.74)] and females [HR 2.19 (95% CI: 1.72–2.78)]; Mexican Americans [HR 1.64 (95% CI: 1.24–2.19)] and non-Hispanic Whites [HR 1.82 (95% CI: 1.45–2.30)]. Regarding education levels, the hazard ratios were [HR 2.13 (95% CI: 1.62–2.80)] for those with less than a 9th-grade education, [HR 1.67 (95% CI: 1.11–2.49)] for those with 9–11 years of education, [HR 1.91 (95% CI: 1.36–2.70)] for high school graduates or those with equivalent education, and [HR 1.92 (95% CI: 1.27–2.89)] for those with some college education or an Associate of Arts (AA) degree. For marital status, the hazard ratios were [HR 1.86 (95% CI: 1.50–2.31)] for married participants and [HR 2.53 (95% CI: 1.93–3.31)] for those with other marital statuses. Among participants without diabetes mellitus (DM), the hazard ratio was [HR 1.88 (95% CI: 1.54–2.28)], while for those with DM, it was [HR 1.74 (95% CI: 1.32–2.31)]; for participants without hypertension, the hazard ratio was [HR 1.60 (95% CI: 1.33–1.91)], and for those with hypertension, it was [HR 3.09 (95% CI: 2.19–4.38)]. For participants without cardiovascular disease, the hazard ratio was [HR 1.71 (95% CI: 1.41–2.07)], while for those with cardiovascular disease, it was [HR 2.04 (95% CI: 1.49–2.77)].

However, no significant associations were found in participants aged 20–60 years, those with college or higher education, those living with a partner, unmarried individuals, and participants of other racial groups. (Subgroup analyses for cardiovascular disease, cancer, and chronic lower respiratory disease are presented in S2A–S2C Table).

**Table 2:** Subgroup of association between systemic inflammatory response index (SIRI) and all-cause mortality.

Subgroup	Q1	Q2 Adjusted HR (95% CI)	*P*	Q3 Adjusted HR (95% CI)	*P*	*P* for interaction
AGE						0.3362
<60	1.0	1.09 (0.70, 1.68)	0.7111	1.18 (0.75, 1.84)	0.4728	
≥60	1.0	1.35 (1.12, 1.61)	0.0013	1.72 (1.45, 2.05)	<0.0001	
GENDER						0.0561
Male	1.0	1.12 (0.89, 1.41)	0.3204	1.40 (1.12, 1.74)	0.0026	
Female	1.0	1.57 (1.24, 2.00)	0.0002	2.19 (1.72, 2.78)	<0.0001	
RACE						0.8699
Mexican American	1.0	1.27 (0.97, 1.67)	0.0871	1.64 (1.24, 2.19)	0.0006	
Other Hispanic	1.0	1.50 (0.74, 3.02)	0.2563	1.73 (0.80, 3.73)	0.1609	
Non-Hispanic White	1.0	1.47 (1.15, 1.88)	0.0018	1.82 (1.45, 2.30)	<0.0001	
Non-Hispanic Black	1.0	1.65 (0.77, 3.52)	0.1969	1.57 (0.77, 3.17)	0.2116	
Other Race Including Multi- Racial	1.0	0.58 (0.19, 1.78)	0.3403	0.81 (0.31, 2.13)	0.6708	
EDUCATION						0.4310
Less Than 9th Grade	1.0	1.27 (0.97, 1.68)	0.0840	2.13 (1.62, 2.80)	<0.0001	
9-11th Grade (Includes 12th grade with no diploma)	1.0	1.37 (0.90, 2.08)	0.1441	1.67 (1.11, 2.49)	0.0128	
High School Grad/GED or Equivalent	1.0	1.52 (1.06, 2.17)	0.0215	1.91 (1.36, 2.70)	0.0002	
Some College or AA degree	1.0	1.80 (1.18, 2.73)	0.0060	1.92 (1.27, 2.89)	0.0018	
College Graduate or above	1.0	1.22 (0.69, 2.15)	0.4982	1.39 (0.82, 2.33)	0.2202	
MARITAL STATUS						0.0015
Married	1.0	1.33 (1.06, 1.67)	0.0122	1.86 (1.50, 2.31)	<0.0001	
Living with partner	1.0	1.16 (0.40, 3.38)	0.7820	0.52 (0.13, 2.05)	0.3531	
Never married	1.0	0.55 (0.26, 1.20)	0.1342	0.44 (0.20, 0.95)	0.0366	
Other	1.0	1.84 (1.40, 2.43)	<0.0001	2.53 (1.93, 3.31)	<0.0001	
DIABETES						0.9183
No	1.0	1.44 (1.18, 1.76)	0.0004	1.88 (1.54, 2.28)	<0.0001	
Yes	1.0	1.39 (1.04, 1.85)	0.0278	1.74 (1.32, 2.31)	0.0001	
HYPERLIPIDEMIA						0.0006
No	1.0	1.20 (0.99, 1.45)	0.0650	1.60 (1.33, 1.91)	<0.0001	
Yes	1.0	2.34 (1.65, 3.30)	<0.0001	3.09 (2.19, 4.38)	<0.0001	
PRECVD						0.3433
No	1.0	1.30 (1.07, 1.58)	0.0085	1.71 (1.41, 2.07)	<0.0001	
Yes	1.0	1.66 (1.20, 2.31)	0.0025	2.04 (1.49, 2.77)	<0.0001	

### 3.4 Sensitivity analyses

In the sensitivity analyses, excluding participants with cardiovascular disease (CVD), Model 1 for the Q3 group showed the following associations: all-cause mortality [HR 1.34 (95% CI: 0.92–1.94)], cardiovascular disease mortality [HR 2.53 (95% CI: 1.65–3.89)], cancer mortality [HR 1.34 (95% CI: 0.92–1.94)], and chronic lower respiratory disease mortality [HR 3.32 (95% CI: 1.32–8.36)]. In Model 3, the Q3 group showed associations with all-cause mortality [HR 1.17 (95% CI: 0.95–1.43)], cardiovascular disease mortality [HR 1.60 (95% CI: 1.02–2.52)], cancer mortality [HR 0.80 (95% CI: 0.54–1.20)], and chronic lower respiratory disease mortality [HR 1.79 (95% CI: 0.68–4.72)]. In the group excluding participants who died within the first two years of follow-up, Model 3 for the Q3 group showed associations with all-cause mortality [HR 1.19 (95% CI: 1.00–1.42)], cardiovascular disease mortality [HR 1.37 (95% CI: 0.96–1.95)], cancer mortality [HR 0.85 (95% CI: 0.58–1.25)], and chronic lower respiratory disease mortality [HR 1.51 (95% CI: 0.68–3.39)]. In the group excluding participants under 50 years of age, Model 3 for the Q3 group showed associations with all-cause mortality [HR 1.26 (95% CI: 1.06–1.50)], cardiovascular disease mortality [HR 1.37 (95% CI: 0.98–1.91)], cancer mortality [HR 0.97 (95% CI: 0.67–1.39)], and chronic lower respiratory disease mortality [HR 1.76 (95% CI: 0.84–3.69)].(Baseline data and sensitivity analyses for CVD, cancer, and chronic lower respiratory disease are presented in S3A, S3B, S4A, S4B, S5A and S5B Tables, respectively, with Kaplan-Meier (KM) curve analyses shown in S1–S3 Figs).

### 3.5 Mortality relationships based on SIRI and sarcopenia status

Table 3. The HRs, 95% CIs, and *p*-values for all-cause mortality across different SIRI and sarcopenia status groups in Model 3 were as follows: low SIRI with sarcopenia: [HR 1.14 (95% CI: 1.00, 1.31)], *p* = 0.051; high SIRI without sarcopenia: [HR 1.31 (95% CI: 1.21, 1.41)], *p*<0.0001; high SIRI with sarcopenia: [HR 1.49 (95% CI: 1.35, 1.65)], *p*<0.0001. For cardiovascular disease mortality: low SIRI with sarcopenia: [HR 1.08 (95% CI: 0.83, 1.41)], *p* = 0.565; high SIRI without sarcopenia: [HR 1.34 (95% CI: 1.15, 1.57)], *p* = 0.0001; high SIRI with sarcopenia: [HR 1.48 (95% CI: 1.22, 1.80)], *p*<0.0001.

**Table 3:** All-cause mortality and cause-specific mortality by systemic inflammatory response index (SIRI) and sarcopenia(SP) status.

All-cause mortality						
	Model 1		Model 2		Model 3	
Group	95%CI	*p*	95%CI	*p*	95%CI	*p*
Low SIRI/SP-	ref		ref		ref	
Low SIRI/SP+	2.37 (2.09, 2.70)	<0.0001	1.28 (1.12, 1.46)	0.0002	1.14 (1.00, 1.31)	0.0506
High SIRI/SP-	1.69 (1.57, 1.81)	<0.0001	1.42 (1.31, 1.53)	<0.0001	1.31 (1.21, 1.41)	<0.0001
HighSIRI/SP+	4.68 (4.27, 5.13)	<0.0001	1.76 (1.60, 1.95)	<0.0001	1.49 (1.35, 1.65)	<0.0001
*P* for trend		<0.001		<0.001		<0.001
Cardiavascular disease mortality						
	Model 1		Model 2		Model 3	
Group	95%CI	*p*	95%CI	*p*	95%CI	*p*
Low SIRI/SP-	ref		ref		ref	
Low SIRI/SP+	2.37 (1.83, 3.05)	<0.0001	1.25 (0.96, 1.63)	0.0949	1.08 (0.83, 1.41)	0.5648
High SIRI/SP-	1.77 (1.53, 2.05)	<0.0001	1.46 (1.25, 1.70)	<0.0001	1.34 (1.15, 1.57)	0.0001
HighSIRI/SP+	5.15 (4.32, 6.15)	<0.0001	1.81 (1.49, 2.19)	<0.0001	1.48 (1.22, 1.80)	<0.0001
P for trend		<0.001		<0.001		0.043
Cancer Diseases mortality						
	Model 1		Model 2		Model 3	
Group	95%CI	*p*	95%CI	*p*	95%CI	*p*
Low SIRI/SP-	ref		ref		ref	
Low SIRI/SP+	2.42 (1.88, 3.13)	<0.0001	1.43 (1.09, 1.86)	0.0095	1.39 (1.06, 1.81)	0.0173
High SIRI/SP-	1.47 (1.26, 1.71)	<0.0001	1.23 (1.05, 1.45)	0.0095	1.15 (0.98, 1.35)	0.0879
HighSIRI/SP+	3.62 (2.96, 4.42)	<0.0001	1.47 (1.18, 1.83)	0.0005	1.35 (1.09, 1.68)	0.0070
*P* for trend		<0.001		0.283		0.639
Respiratory diseases mortality						
	Model 1		Model 2		Model 3	
Group	95%CI	*p*	95%CI	*p*	95%CI	*p*
Low SIRI/SP-	ref		ref		ref	
Low SIRI/SP+	2.20 (1.20, 4.03)	0.0104	1.18 (0.64, 2.19)	0.5984	1.00 (0.54, 1.86)	0.9914
High SIRI/SP-	2.30 (1.67, 3.17)	<0.0001	1.66 (1.19, 2.31)	0.0026	1.47 (1.05, 2.06)	0.0237
HighSIRI/SP+	5.25 (3.49, 7.90)	<0.0001	1.68 (1.09, 2.60)	0.0187	1.46 (0.94, 2.26)	0.0940
*P* for trend		<0.001		0.113		0.309

For cancer mortality: low SIRI with sarcopenia: [HR 1.39 (95% CI: 1.06, 1.81)], *p* = 0.017; high SIRI without sarcopenia: [HR 1.15 (95% CI: 0.98, 1.35)], *p* = 0.088; high SIRI with sarcopenia: [HR 1.35 (95% CI: 1.09, 1.68)], *p* = 0.007. For chronic lower respiratory disease mortality: low SIRI with sarcopenia: [HR 1.00 (95% CI: 0.54, 1.86)], *p* = 0.991; high SIRI without sarcopenia: [HR 1.47 (95% CI: 1.05, 2.06)], *p* = 0.024; high SIRI with sarcopenia: [HR 1.46 (95% CI: 0.94, 2.26)], *p* = 0.094. The results from Model 3 indicate that high SIRI and sarcopenia status significantly increased the risk of all-cause mortality, cardiovascular disease mortality, and cancer mortality. Particularly, individuals with high SIRI and sarcopenia had the highest mortality risk.

## 4. Discussion

Over the past decade, the global incidence of sarcopenia has shown a significant upward trend, largely attributed to population aging, lifestyle changes, and advancements in diagnostic technologies. It is projected that the prevalence of sarcopenia among the elderly population in Europe will increase from 11.1%-20.2% in 2016 to 12.9%-22.3% by 2045 [16]. Numerous studies have demonstrated that inflammation plays a critical role in the aging process of skeletal muscle, contributing to the development of sarcopenia and its complications, and significantly impacting patient mortality [17]. This study analyzes the relationship between the systemic inflammatory response index (SIRI) and all-cause mortality, cardiovascular disease mortality, cancer mortality, and chronic lower respiratory disease mortality using data from the 1999–2018 cycles of the National Health and Nutrition Examination Survey (NHANES). The results indicate that, firstly, in univariate analysis, SIRI is significantly associated with these mortality rates. After adjusting for all covariates, SIRI remains significantly associated with all-cause and cardiovascular disease mortality, with higher SIRI levels generally corresponding to an increased risk of mortality. Subgroup and sensitivity analyses further corroborate these findings. Secondly, smooth curve fitting analysis reveals a nonlinear relationship between SIRI and these mortality rates. Additionally, analysis of the relationship between SIRI and mortality based on different sarcopenia statuses shows that the impact of SIRI on all-cause and cardiovascular disease mortality is greater in the presence of sarcopenia than in its absence.

This study, using NHANES data, found that SIRI is significantly associated with all-cause mortality and cardiovascular disease mortality in sarcopenia patients across three adjusted models, indicating that the correlation between SIRI and these mortality outcomes is not confounded by other covariates. Moreover, Model 3 demonstrated that, compared to the Q1 level, the Q3 level of SIRI was associated with a 0.24-fold increase in all-cause mortality and a 0.46-fold increase in cardiovascular disease mortality among sarcopenia patients. Additionally, subgroup analysis in this study revealed that elevated SIRI significantly increased the risk of all-cause mortality regardless of gender, age, diabetes status, or presence of cardiovascular disease. Similarly, Jin et al. found that elevated SIRI significantly increased the risk of all-cause mortality, and this association was consistent across different age groups, genders, and whether or not the individuals had diabetes or cardiovascular disease [18]. Xia et al. discovered that elevated SIRI levels were associated with an increased risk of all-cause mortality among U.S. adults, with similar consistent results across gender- and age-based subgroup analyses [19]. Our findings are consistent with previous research.

Furthermore, this study, through subgroup analysis, found that the impact of SIRI on mortality rates varies among different populations, with non-Hispanic Whites and Mexican Americans exhibiting significantly higher mortality rates at elevated SIRI levels compared to other racial groups. Similarly, Crews et al. observed that while there were no racial differences in mortality among dialysis patients at low inflammation levels, significant racial disparities emerged at higher inflammation levels, partially explaining our findings [20]. Differences in genetics, culture, and lifestyle among racial groups may lead to varying sensitivities to inflammation, suggesting that although SIRI is a useful indicator of inflammation and health risk, its effects may be modulated by differences in health status, lifestyle, social support, and racial background in specific subgroups [21–23].

To further validate the association between SIRI and mortality in sarcopenia patients, this study conducted three sensitivity analyses. The results demonstrated that after excluding patients with cardiovascular disease, SIRI remained significantly associated with cardiovascular disease mortality, indicating that it is an independent predictor of cardiovascular disease mortality. After excluding patients who died within two years of follow-up, the association between SIRI and all-cause mortality approached significance, suggesting that early mortality may have influenced the results, although the overall trend remained consistent. After excluding individuals under 50 years of age, the analysis showed that SIRI was significantly associated with all-cause mortality and nearly significantly associated with cardiovascular disease mortality, implying that in the elderly population, cardiovascular risk prediction may require the consideration of additional factors beyond SIRI to more accurately assess the risk.

Previous studies have shown that the inflammatory response in sarcopenia patients is often the result of multiple factors. Firstly, as individuals age, the body gradually enters a state of low-grade chronic inflammation, commonly referred to as “inflammaging” [24]. Research on chronic low-grade inflammation in the elderly has demonstrated that inflammatory cytokines such as IL-6 and TNF-α are significantly elevated in sarcopenia patients. These factors lead to muscle mass decline by activating protein degradation pathways and inhibiting protein synthesis [25]. Additionally, obesity and metabolic syndrome, which are prevalent among the elderly, are often accompanied by chronic inflammation of adipose tissue. In obesity and age-related sarcopenia, the inflammatory state of adipose tissue exacerbates muscle inflammation through the secretion of various cytokines and chemokines [26]. This inflammation not only affects local muscle tissue but also contributes to systemic low-grade inflammation, as seen in lipotoxic environments and insulin resistance, further deteriorating muscle function [27]. Furthermore, with advancing age, mitochondrial dysfunction can lead to excessive production of reactive oxygen species (ROS), triggering oxidative damage and inflammatory responses [28]. Oxidative stress and molecular inflammation play critical roles in age-related muscle atrophy. These factors disrupt the balance between protein synthesis and degradation, lead to mitochondrial dysfunction, and induce apoptosis, ultimately resulting in sarcopenia [29, 30]. Lastly, recent studies have revealed a close link between gut microbiota dysbiosis and systemic inflammation. Impaired gut barrier function can allow bacterial toxins, such as lipopolysaccharides, to permeate and activate systemic inflammatory responses. In sarcopenia patients, gut microbiota dysbiosis may be a significant source of the pro-inflammatory environment, further exacerbating muscle degradation [31]. Moreover, numerous studies have found that inflammatory markers detected in blood tests, including the Systemic Immune-Inflammation Index (SII), C-reactive protein (CRP), and interleukin-17, are closely associated with sarcopenia [32–34]. These markers are typically elevated in sarcopenia patients and are correlated with patient prognosis. The elevation of these markers indicates a systemic inflammatory state, consistent with the role of SIRI as a comprehensive inflammation index.

Our study findings indicate that SIRI is significantly associated with all-cause and cardiovascular disease mortality in sarcopenia patients and also impacts cancer and chronic lower respiratory disease mortality. As an index reflecting the ratio of neutrophils, monocytes, and lymphocytes, SIRI encapsulates the combined effects of inflammation and immune dysfunction. A high SIRI value signifies a high inflammatory burden and a low immune defense status. Existing research suggests that the impact of SIRI on sarcopenia may be attributed to enzymes and reactive oxygen species (ROS) released by neutrophils, which promote inflammatory responses by damaging vascular endothelial cells and accelerating the progression of atherosclerosis, potentially increasing the risk of thrombosis and cardiovascular events [35–40]. Lymphocytes play a crucial role in regulating the body’s immune response, with T lymphocytes and B lymphocytes being key in controlling inflammation and preventing disease progression [41–44]. T cells, particularly CD8+ cytotoxic T cells, are capable of eliminating infected or cancerous cells and preventing the spread of inflammation [45]. However, in an immunosuppressive state, such as lymphopenia, chronic low-grade inflammation can develop, which is detrimental to muscle tissue and may further damage muscle through the activation of inflammation-related pathways, such as the NF-κB and JAK/STAT pathways [46, 47]. B cells are responsible for antibody production and are involved in humoral immunity. Lymphopenia may result in insufficient antibody production, making sarcopenia patients more susceptible to infections. Recurrent infections not only increase the systemic inflammatory burden but also accelerate muscle function decline by inducing acute-phase responses, such as elevated CRP [48].

Targeting SIRI through interventions such as anti-inflammatory therapies, antioxidants, nutrition, and exercise may help in managing sarcopenia. Although NSAIDs are effective, their use in the elderly must be approached with caution due to potential side effects [49]. Antioxidants like vitamins C and E, as well as glutathione, can alleviate oxidative stress and indirectly lower SIRI levels [50–52]. Nutrition, particularly diets rich in protein, essential amino acids, and omega-3 fatty acids, can mitigate the inflammatory impact on muscle [53]. Regular exercise, including both aerobic and resistance training, not only enhances muscle function but also reduces inflammation and SIRI levels [54, 55]. Further research could explore specific exercise regimens and their long-term efficacy in sarcopenia management.

The strengths of this study lie in its high-quality data and large sample size, with NHANES data collected by professionals. The robustness of the findings has been validated through various methods. However, there are several limitations to the study: Firstly, due to the constraints of the NHANES database, our analysis did not account for participants’ medication use, thereby omitting critical data on drugs such as adriamycin or naproxen, which may elevate or lower inflammation levels. Consequently, our findings may not fully reflect the actual situation. Future research should consider more potential confounding factors to enhance the external validity of the results. Secondly, participants may have lost follow-up opportunities during the study, which could lead to incomplete data and potential bias. Future studies should employ longitudinal designs and more comprehensive data collection to further validate the association between SIRI and mortality in sarcopenia patients, while also taking into account factors such as race, diet, and lifestyle. Lastly, since NHANES data primarily originates from the U.S. population, although the sample size is large and diverse, the applicability of the results to other racial or regional populations requires further verification. Future research should be conducted in diverse populations to evaluate the clinical utility of SIRI across different racial and cultural contexts.

## 5. Conclusion

This study demonstrates that SIRI is significantly associated with all-cause and cardiovascular disease mortality in sarcopenia patients aged 20 years and older, highlighting the critical role of inflammation in the prognosis of sarcopenia patients. Future research should further explore the mechanisms of inflammation in sarcopenia and develop targeted interventions, such as nutritional adjustments, physical activity, psychological interventions, and pharmacological treatments.

## Supporting information

S1 Fig Kaplan-Meier curves showing differences in all-cause and cause-specific mortality for SIRI, excluding initial cardiovascular disease. (TIF)

S2 Fig Kaplan-Meier curves showing differences in all-cause and cause-specific mortality for SIRI, excluding participants who died within two years. (TIF)

S3 Fig Kaplan-Meier curves showing differences in all-cause and cause-specific mortality for SIRI, excluding participants under 50 years of age. (TIF)

S1 Table Association of SIRI with all-cause and cause-specific mortality in sarcopenia participants. (DOCX)

S2 Table A. Subgroup of association between systemic inflammatory response index (SIRI) and cardiovascular disease mortality. B. Subgroup of association between systemic inflammatory response index (SIRI) and cancer mortality. C. Subgroup of association between systemic inflammatory response index (SIRI) and chronic lower respiratory disease mortality.
(ZIP)

S3 Table A. Characteristics of sarcopenia: participants without pre-existing CVD at baseline in the NHANES study. B. Association of SIRI with all-cause and cause-specific mortality in sarcopenia participants without pre-existing CVD at baseline.
(ZIP)

S4 Table A. Characteristics of sarcopenia: excluding participants who died within two years in the NHANES study. B. Association of SIRI with all-cause and cause-specific mortality in sarcopenia participants (excluding participants who died within two years).
(ZIP)

S5 Table A. Characteristics of sarcopenia participants in the NHANES (excluding participants under the age of 50). B. Association of SIRI with all-Cause and cause-specific mortality in sarcopenia participants(excluding participants under the age of 50).
(ZIP)

S1 Appendix Original data. (XLSX)
