# Supplementary figures and images for: Association between the systemic inflammatory response index and mortality in patients with sarcopenia
Source: PLoS One. 2024 Nov 18;19(11):e0312383. doi: 10.1371/journal.pone.0312383 (PMC11573146; doi:10.1371/journal.pone.0312383)

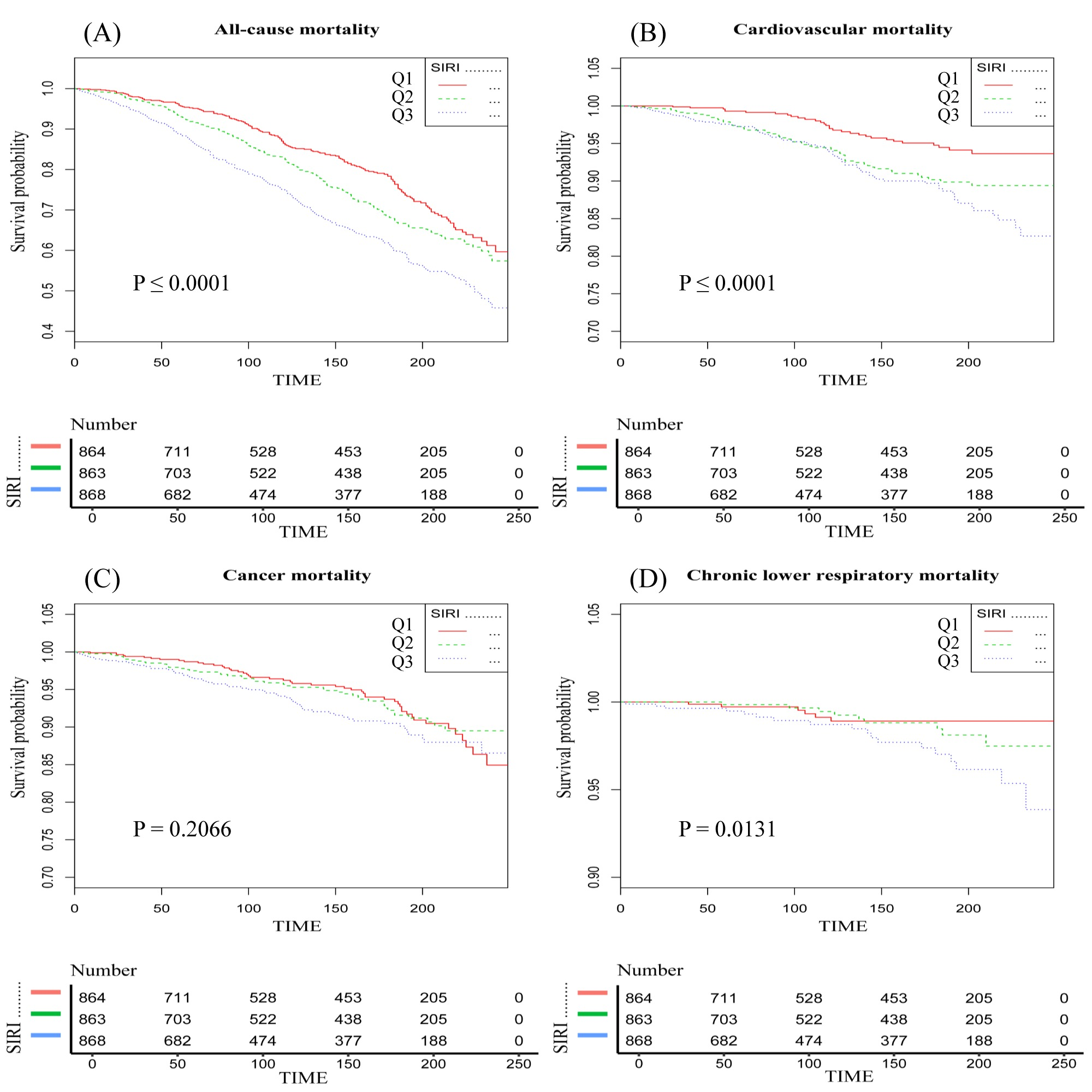

Supplement: S1 Fig — (TIF) [file pone.0312383.s001.tif]

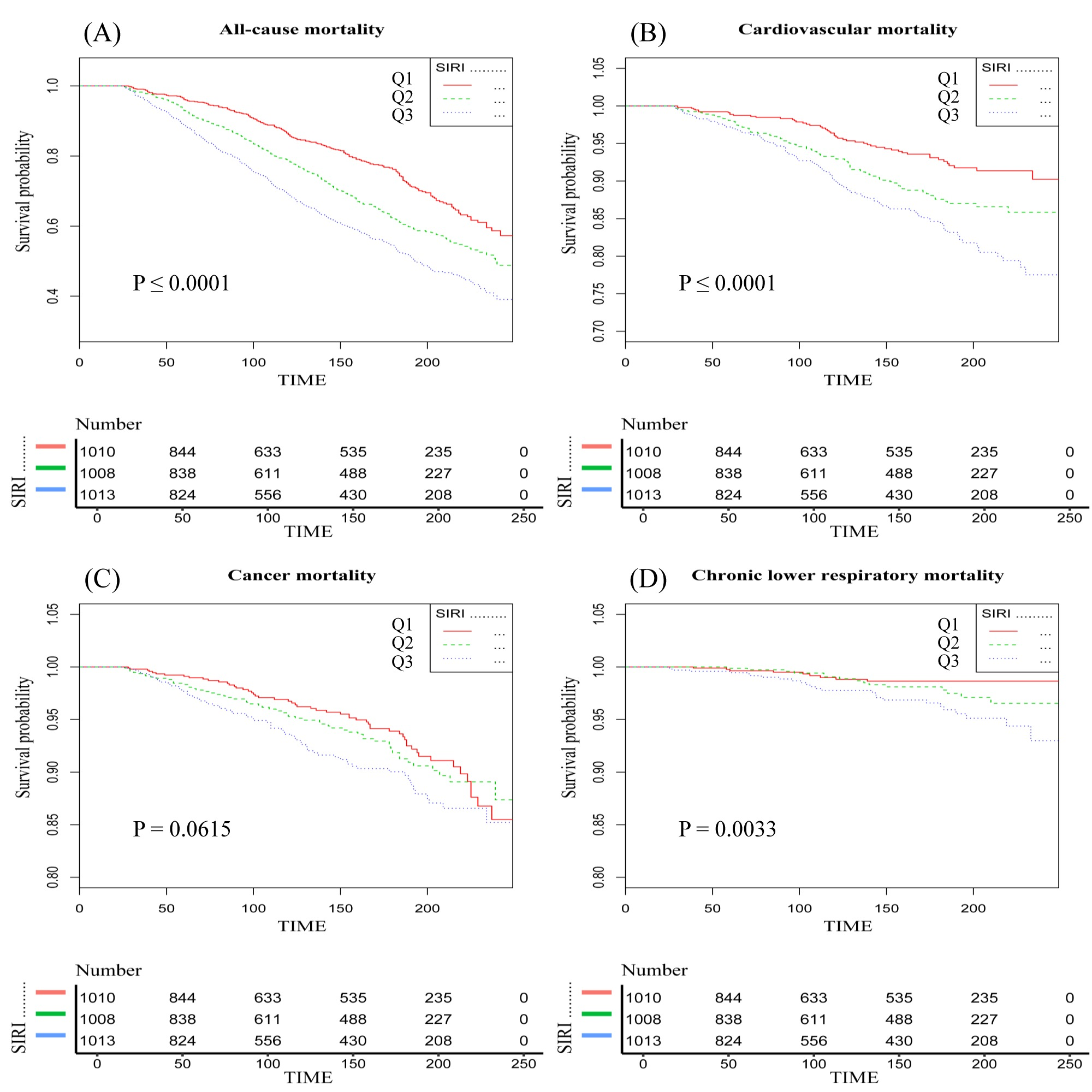

Supplement: S2 Fig — (TIF) [file pone.0312383.s002.tif]

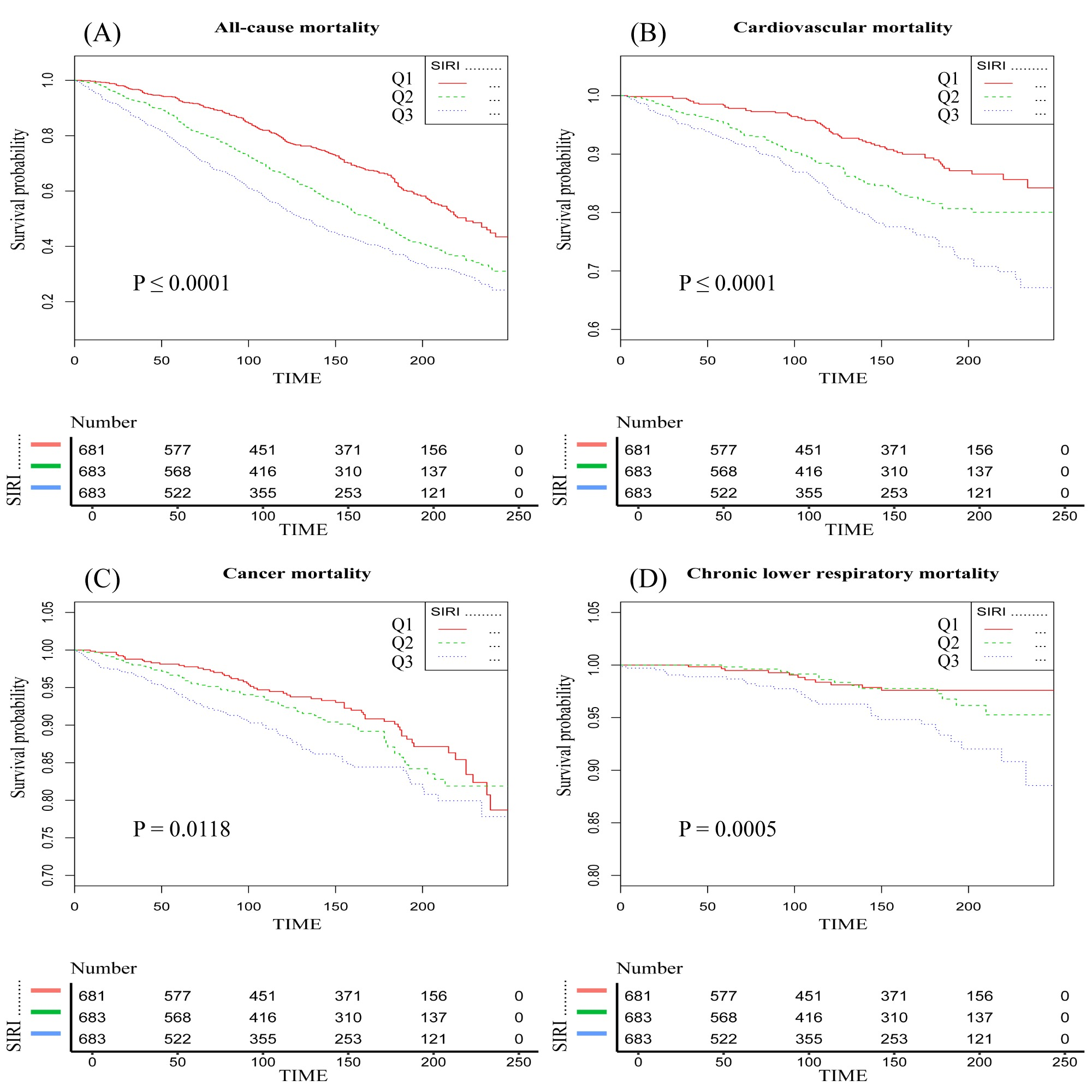

Supplement: S3 Fig — (TIF) [file pone.0312383.s003.tif]
