# Supplementary material for: Association between the systemic inflammatory response index and mortality in patients with sarcopenia
Source: PLoS One. 2024 Nov 18;19(11):e0312383. doi: 10.1371/journal.pone.0312383 (PMC11573146; doi:10.1371/journal.pone.0312383)
Supplement: S1 Table — (DOCX) [file pone.0312383.s004.docx]

Table S1 Association of SIRI with All-Cause and Cause-Specific Mortality in Sarcopenia Participants.

|  | All-cause mortality | | | | | |
| --- | --- | --- | --- | --- | --- | --- |
|  | Model 1 | | Model 2 | | Model 3 | |
| Character | 95%CI | *p* | 95%CI | *p* | 95%CI | *p* |
| Q1 | ref |  | ref |  | ref |  |
| Q2 | 1.57 (1.33, 1.84) | <0.0001 | 1.22 (1.04, 1.45) | 0.0174 | 1.15 (0.97, 1.36) | 0.1089 |
| Q3 | 2.24 (1.92, 2.62) | <0.0001 | 1.37 (1.16, 1.61) | 0.0002 | 1.24 (1.05, 1.47) | 0.0104 |
| P for trend |  | <0.001 |  | 0.015 |  | 0.370 |
|  | Cardiovascular disease mortality | | | | | |
|  | Model 1 | | Model 2 | | Model 3 | |
| Character | 95%CI | *p* | 95%CI | *p* | 95%CI | *p* |
| Q1 | ref |  | ref |  | ref |  |
| Q2 | 1.85 (1.32, 2.57) | 0.0003 | 1.38 (0.98, 1.93) | 0.0635 | 1.28 (0.90, 1.80) | 0.1674 |
| Q3 | 2.87 (2.10, 3.93) | <0.0001 | 1.63 (1.17, 2.26) | 0.0036 | 1.46 (1.04, 2.04) | 0.0278 |
| P for trend |  | <0.001 |  | 0.074 |  | 0.358 |
|  | Cancer Diseases mortality | | | | | |
|  | Model 1 | | Model 2 | | Model 3 | |
| Character | 95%CI | *p* | 95%CI | *p* | 95%CI | *p* |
| Q1 | ref |  | ref |  | ref |  |
| Q2 | 1.21 (0.86, 1.71) | 0.2774 | 0.93 (0.66, 1.33) | 0.7015 | 0.90 (0.63, 1.28) | 0.5550 |
| Q3 | 1.71 (1.23, 2.37) | 0.0013 | 1.03 (0.73, 1.45) | 0.8872 | 0.97 (0.68, 1.37) | 0.8480 |
| P for trend |  | 0.017 |  | 0.471 |  | 0.322 |
|  | Respiratory diseases mortality | | | | | |
|  | Model 1 | | Model 2 | | Model 3 | |
| Character | 95%CI | *p* | 95%CI | *p* | 95%CI | *p* |
| Q1 | ref |  | ref |  | ref |  |
| Q2 | 1.51 (0.67, 3.40) | 0.3181 | 1.06 (0.47, 2.43) | 0.8857 | 0.97 (0.42, 2.24) | 0.9356 |
| Q3 | 3.48 (1.70, 7.15) | 0.0007 | 1.80 (0.85, 3.83) | 0.1269 | 1.63 (0.75, 3.51) | 0.2154 |
| P for trend |  | 0.002 |  | 0.113 |  | 0.309 |

Model 1: No adjustment for covariates. Model 2: adjusted for age, gender, and race. Model 3: Age, gender, race, education, household income to poverty ratio, marital status, smoking status, drinking status, diabetes, hypertension, hyperlipidemia, UACR, ALT, and AST.
