# Supplementary material for: Association between the systemic inflammatory response index and mortality in patients with sarcopenia
Source: PLoS One. 2024 Nov 18;19(11):e0312383. doi: 10.1371/journal.pone.0312383 (PMC11573146; doi:10.1371/journal.pone.0312383)
Supplement: S2 Table — A. Subgroup of association between systemic inflammatory response index (SIRI) and cardiovascular disease mortality. B. Subgroup of association between systemic inflammatory response index (SIRI) and cancer mortality. C. Subgroup of association between systemic inflammatory response index (SIRI) and chronic lower respiratory disease mortality. (ZIP) [file pone.0312383.s005.zip › S2A_Table.docx]

Table S2A Subgroup of association between systemic inflammatory response index (SIRI) and Cardiovascular disease mortality.

| Subgroup | Q1 | Q2 Adjusted  HR (95% CI) | *P* | Q3 Adjusted  HR (95% CI) | *P* | *P* for interaction |
| --- | --- | --- | --- | --- | --- | --- |
| AGE |  |  |  |  |  | 0.9311 |
| <60 | 1.0 | 1.43 (0.53, 3.87) | 0.4864 | 1.60 (0.59, 4.31) | 0.3521 |  |
| ≥60 | 1.0 | 1.50 (1.04, 2.14) | 0.0281 | 2.03 (1.44, 2.86) | <0.0001 |  |
| GENDER |  |  |  |  |  | 0.0733 |
| Male | 1.0 | 1.02 (0.64, 1.62) | 0.9280 | 1.41 (0.91, 2.17) | 0.1211 |  |
| Female | 1.0 | 2.22 (1.36, 3.62) | 0.0014 | 3.13 (1.92, 5.10) | <0.0001 |  |
| RACE |  |  |  |  |  | 0.9144 |
| Mexican American | 1.0 | 1.67 (0.94, 2.96) | 0.0792 | 1.80 (0.98, 3.32) | 0.0590 |  |
| Other Hispanic | 1.0 | 0.38 (0.02, 7.55) | 0.5292 | 6.75 (0.49, 92.76) | 0.1532 |  |
| Non-Hispanic White | 1.0 | 1.42 (0.90, 2.26) | 0.1349 | 2.04 (1.33, 3.15) | 0.0012 |  |
| Non-Hispanic Black | 1.0 | 1.77 (0.36, 8.79) | 0.4835 | 1.16 (0.24, 5.61) | 0.8504 |  |
| Other Race Including Multi- Racial | 1.0 | 0.37 (0.02, 6.82) | 0.5051 | 0.90 (0.09, 8.77) | 0.9309 |  |
| EDUCATION |  |  |  |  |  | 0.4093 |
| Less Than 9th Grade | 1.0 | 1.71 (0.98, 3.00) | 0.0612 | 2.73 (1.55, 4.79) | 0.0005 |  |
| 9-11th Grade (Includes 12th grade with no diploma) | 1.0 | 0.64 (0.25, 1.62) | 0.3461 | 1.40 (0.65, 3.00) | 0.3882 |  |
| High School Grad/GED or Equivalent | 1.0 | 2.86 (1.26, 6.49) | 0.0122 | 3.26 (1.46, 7.31) | 0.0040 |  |
| Some College or AA degree | 1.0 | 1.51 (0.64, 3.56) | 0.3451 | 1.98 (0.88, 4.48) | 0.1009 |  |
| College Graduate or above | 1.0 | 1.56 (0.60, 4.06) | 0.3634 | 1.51 (0.62, 3.70) | 0.3671 |  |
| MARITAL STATUS |  |  |  |  |  | 0.6711 |
| Married | 1.0 | 1.60 (1.00, 2.56) | 0.0484 | 2.37 (1.51, 3.72) | 0.0002 |  |
| Living with partner | 1.0 | 0.23 (0.01, 4.27) | 0.3231 | 0.25 (0.02, 3.42) | 0.2999 |  |
| Never married | 1.0 | 0.55 (0.04, 7.86) | 0.6598 | 2.32 (0.34, 15.66) | 0.3888 |  |
| Other | 1.0 | 1.99 (1.18, 3.38) | 0.0105 | 2.71 (1.63, 4.51) | 0.0001 |  |
| DIABETES |  |  |  |  |  | 0.4505 |
| No | 1.0 | 1.85 (1.22, 2.79) | 0.0037 | 2.24 (1.49, 3.37) | <0.0001 |  |
| Yes | 1.0 | 1.30 (0.73, 2.33) | 0.3788 | 2.32 (1.35, 3.99) | 0.0024 |  |
| HYPERLIPIDEMIA |  |  |  |  |  | 0.0015 |
| No | 1.0 | 1.49 (1.03, 2.16) | 0.0344 | 1.70 (1.18, 2.44) | 0.0043 |  |
| Yes | 1.0 | 2.15 (0.96, 4.81) | 0.0624 | 5.83 (2.76, 12.30) | <0.0001 |  |

Table S2A Continued

| Subgroup | Q1 | Q2 Adjusted  HR (95% CI) | *P* | Q3 Adjusted  HR (95% CI) | *P* | *P* for interaction |
| --- | --- | --- | --- | --- | --- | --- |
| PRECVD |  |  |  |  |  | 0.3940 |
| No | 1.0 | 1.91 (1.23, 2.96) | 0.0038 | 2.46 (1.59, 3.80) | <0.0001 |  |
| Yes | 1.0 | 1.13 (0.66, 1.94) | 0.6542 | 1.65 (1.01, 2.70) | 0.0444 |  |

Each stratum was adjusted for household income and poverty rate, smoking status, alcohol drinkers, aspartate aminotransferase, alanine aminotransferase, urinary albumin creatinine ratio, and hypertension.
