# Supplementary material for: Association between the systemic inflammatory response index and mortality in patients with sarcopenia
Source: PLoS One. 2024 Nov 18;19(11):e0312383. doi: 10.1371/journal.pone.0312383 (PMC11573146; doi:10.1371/journal.pone.0312383)
Supplement: S3 Table — A. Characteristics of sarcopenia: participants without pre-existing CVD at baseline in the NHANES study. B. Association of SIRI with all-cause and cause-specific mortality in sarcopenia participants without pre-existing CVD at baseline. (ZIP) [file pone.0312383.s006.zip › S3A_Table.docx]

Table S3A Characteristics of Sarcopenia: Participants Without Pre-existing CVD at Baseline in the NHANES Study.

| Variables | Q1(n=864) | Q2(n=863) | Q3(n=868) | *P*value |
| --- | --- | --- | --- | --- |
| AGE | 52.32 ± 15.51 | 53.08 ± 17.08 | 55.44 ± 18.37 | <0.001 |
| ALT | 29.12 ± 18.95 | 29.18 ± 22.42 | 28.29 ± 19.79 | 0.597 |
| AST | 26.63 ± 12.17 | 26.03 ± 13.06 | 25.85 ± 12.69 | 0.410 |
| UACR | 39.66 ± 192.71 | 72.69 ± 472.68 | 61.74 ± 261.46 | 0.108 |
| SIRI | 0.65 ± 0.16 | 1.12 ± 0.15 | 2.24 ± 1.16 | <0.001 |
| GENDER |  |  |  | <0.001 |
| Male | 352 (40.74%) | 422 (48.90%) | 528 (60.83%) |  |
| Female | 512 (59.26%) | 441 (51.10%) | 340 (39.17%) |  |
| RACE |  |  |  | <0.001 |
| Mexican American | 437 (50.58%) | 398 (46.12%) | 313 (36.06%) |  |
| Other Hispanic | 75 (8.68%) | 82 (9.50%) | 75 (8.64%) |  |
| Non-Hispanic White | 206 (23.84%) | 286 (33.14%) | 387 (44.59%) |  |
| Non-Hispanic Black | 51 (5.90%) | 28 (3.24%) | 43 (4.95%) |  |
| Other Race Including Multi-Racial | 95 (11.00%) | 69 (8.00%) | 50 (5.76%) |  |
| EDUCATION |  |  |  | 0.014 |
| Less Than 9th Grade | 257 (29.75%) | 274 (31.75%) | 217 (25.00%) |  |
| 9-11th Grade (Includes 12th grade with no diploma) | 155 (17.94%) | 136 (15.76%) | 137 (15.78%) |  |
| High School Grad/GED or Equivalent | 198 (22.92%) | 178 (20.63%) | 200 (23.04%) |  |
| Some College or AA degree | 154 (17.82%) | 185 (21.44%) | 205 (23.62%) |  |
| College Graduate or above | 100 (11.57%) | 90 (10.43%) | 109 (12.56%) |  |
| MARITAL STATUS |  |  |  | <0.001 |
| Married | 523 (60.53%) | 522 (60.49%) | 489 (56.34%) |  |
| Living with partner | 51 (5.90%) | 53 (6.14%) | 47 (5.41%) |  |
| Never married | 99 (11.46%) | 87 (10.08%) | 140 (16.13%) |  |
| Other | 191 (22.11%) | 201 (23.29%) | 192 (22.12%) |  |
| PIR |  |  |  | 0.949 |
| High | 171 (19.79%) | 163 (18.89%) | 163 (18.78%) |  |
| Medium | 375 (43.40%) | 380 (44.03%) | 392 (45.16%) |  |
| Low | 318 (36.81%) | 320 (37.08%) | 313 (36.06%) |  |
| SMOKE |  |  |  | <0.001 |
| Never | 551 (63.77%) | 509 (58.98%) | 435 (50.12%) |  |

Table 1 Continued

| Variables | Q1(n=1047) | Q2(n=1046) | Q3(n=1048) | *P*value |
| --- | --- | --- | --- | --- |
| Former | 215 (24.88%) | 214 (24.80%) | 256 (29.49%) |  |
| Now | 98 (11.34%) | 140 (16.22%) | 177 (20.39%) |  |
| ALCOHOL USE |  |  |  | <0.001 |
| Never | 199 (23.03%) | 172 (19.93%) | 132 (15.21%) |  |
| Former | 140 (16.20%) | 122 (14.14%) | 118 (13.59%) |  |
| Mild | 273 (31.60%) | 302 (34.99%) | 330 (38.02%) |  |
| Moderate | 111 (12.85%) | 101 (11.70%) | 130 (14.98%) |  |
| Heavy | 141 (16.32%) | 166 (19.24%) | 158 (18.20%) |  |
| DIABETES |  |  |  | 0.356 |
| No | 679 (78.59%) | 664 (76.94%) | 657 (75.69%) |  |
| Yes | 185 (21.41%) | 199 (23.06%) | 211 (24.31%) |  |
| HYPERLIPIDEMIA |  |  |  | 0.019 |
| No | 599 (69.33%) | 618 (71.61%) | 654 (75.35%) |  |
| Yes | 265 (30.67%) | 245 (28.39%) | 214 (24.65%) |  |
| HYPERTENSION |  |  |  | <0.001 |
| No | 593 (68.63%) | 545 (63.15%) | 504 (58.06%) |  |
| Yes | 271 (31.37%) | 318 (36.85%) | 364 (41.94%) |  |

ALT: alanine aminotransferase; AST: aspartate aminotransferase; UACR: urine albumin to creatinine ratio; SIRI: systemic immune-inflammation index;
