# Supplementary material for: Association between the systemic inflammatory response index and mortality in patients with sarcopenia
Source: PLoS One. 2024 Nov 18;19(11):e0312383. doi: 10.1371/journal.pone.0312383 (PMC11573146; doi:10.1371/journal.pone.0312383)
Supplement: S3 Table — A. Characteristics of sarcopenia: participants without pre-existing CVD at baseline in the NHANES study. B. Association of SIRI with all-cause and cause-specific mortality in sarcopenia participants without pre-existing CVD at baseline. (ZIP) [file pone.0312383.s006.zip › S3B_Table.docx]

Table S3B Association of SIRI with All-Cause and Cause-Specific Mortality in Sarcopenia Participants Without Pre-existing CVD at Baseline.

|  | All-cause mortality | | | | | |
| --- | --- | --- | --- | --- | --- | --- |
|  | Model 1 | | Model 2 | | Model 3 | |
| Character | 95%CI | *p* | 95%CI | *p* | 95%CI | *p* |
| Q1 | ref |  | ref |  | ref |  |
| Q2 | 1.29 (1.06, 1.57) | 0.0115 | 1.16 (0.95, 1.42)) | 0.1423 | 1.10 (0.90, 1.35) | 0.3547 |
| Q3 | 1.82 (1.51, 2.20) | <0.0001 | 1.26 (1.04, 1.53) | 0.0212 | 1.17 (0.95, 1.43) | 0.1312 |
|  | Cardiovascular disease mortality | | | | | |
|  | Model 1 | | Model 2 | | Model 3 | |
| Character | 95%CI | *p* | 95%CI | *p* | 95%CI | *p* |
| Q1 | ref |  | ref |  | ref |  |
| Q2 | 1.97 (1.26, 3.05) | 0.0027 | 1.78 (1.14, 2.77) | 0.0116 | 1.66 (1.05, 2.62) | 0.0297 |
| Q3 | 2.53 (1.65, 3.89) | <0.0001 | 1.73 (1.11, 2.70) | 0.0159 | 1.60 (1.02, 2.52) | 0.0402 |
|  | Cancer Diseases mortality | | | | | |
|  | Model 1 | | Model 2 | | Model 3 | |
| Character | 95%CI | *p* | 95%CI | *p* | 95%CI | *p* |
| Q1 | ref |  | ref |  | ref |  |
| Q2 | 1.00 (0.68, 1.49) | 0.9873 | 0.86 (0.57, 1.28) | 0.4524 | 0.84 (0.56, 1.26) | 0.3970 |
| Q3 | 1.34 (0.92, 1.94) | 0.1290 | 0.85 (0.58, 1.26) | 0.4266 | 0.80 (0.54, 1.20) | 0.2856 |
|  | Respiratory diseases mortality | | | | | |
|  | Model 1 | | Model 2 | | Model 3 | |
| Character | 95%CI | *p* | 95%CI | *p* | 95%CI | *p* |
| Q1 | ref |  | ref |  | ref |  |
| Q2 | 1.53 (0.55, 4.31) | 0.4168 | 1.19 (0.42, 3.39) | 0.7418 | 1.02 (0.35, 2.99) | 0.9669 |
| Q3 | 3.32 (1.32, 8.36) | 0.0110 | 1.84 (0.70, 4.84) | 0.2139 | 1.79 (0.68, 4.72) | 0.2417 |

Model 1: No adjustment for covariates. Model 2: adjusted for age, gender, and race. Model 3: Age, gender, race, education, household income to poverty ratio, marital status, smoking status, drinking status, diabetes, hypertension, hyperlipidemia, UACR, ALT, and AST.
