# Supplementary material for: Association between the systemic inflammatory response index and mortality in patients with sarcopenia
Source: PLoS One. 2024 Nov 18;19(11):e0312383. doi: 10.1371/journal.pone.0312383 (PMC11573146; doi:10.1371/journal.pone.0312383)
Supplement: S4 Table — A. Characteristics of sarcopenia: excluding participants who died within two years in the NHANES study. B. Association of SIRI with all-cause and cause-specific mortality in sarcopenia participants (excluding participants who died within two years). (ZIP) [file pone.0312383.s007.zip › S4A_Table.docx]

Table S4A Characteristics of Sarcopenia: Excluding Participants who Died within Two Years in the NHANES Study.

| Variables | Q1(n=1010) | Q2(n=1008) | Q3(n=1013) | *P*value |
| --- | --- | --- | --- | --- |
| AGE | 53.13 ± 15.50 | 55.02 ± 17.31 | 58.11 ± 18.14 | <0.001 |
| ALT | 28.87 ± 18.76 | 27.76 ± 20.53 | 27.91 ± 20.73 | 0.402 |
| AST | 26.68 ± 12.70 | 25.53 ± 12.06 | 25.92 ± 14.75 | 0.141 |
| UACR | 54.02 ± 552.44 | 77.45 ± 460.21 | 79.09 ± 304.71 | 0.376 |
| SIRI | 0.67 ± 0.16 | 1.17 ± 0.16 | 2.37 ± 1.21 | <0.001 |
| GENDER |  |  |  | <0.001 |
| Male | 409 (40.50%) | 511 (50.69%) | 617 (60.91%) |  |
| Female | 601 (59.50%) | 497 (49.31%) | 396 (39.09%) |  |
| RACE |  |  |  | <0.001 |
| Mexican American | 497 (49.21%) | 436 (43.25%) | 326 (32.18%) |  |
| Other Hispanic | 89 (8.81%) | 94 (9.33%) | 78 (7.70%) |  |
| Non-Hispanic White | 256 (25.35%) | 377 (37.40%) | 503 (49.65%) |  |
| Non-Hispanic Black | 64 (6.34%) | 30 (2.98%) | 51 (5.03%) |  |
| Other Race Including Multi-Racial | 104 (10.30%) | 71 (7.04%) | 55 (5.43%) |  |
| EDUCATION |  |  |  | 0.004 |
| Less Than 9th Grade | 299 (29.60%) | 317 (31.45%) | 238 (23.49%) |  |
| 9-11th Grade (Includes 12th grade with no diploma) | 183 (18.12%) | 159 (15.77%) | 176 (17.37%) |  |
| High School Grad/GED or Equivalent | 226 (22.38%) | 216 (21.43%) | 246 (24.28%) |  |
| Some College or AA degree | 188 (18.61%) | 217 (21.53%) | 229 (22.61%) |  |
| College Graduate or above | 114 (11.29%) | 99 (9.82%) | 124 (12.24%) |  |
| MARITAL STATUS |  |  |  | 0.077 |
| Married | 608 (60.20%) | 609 (60.42%) | 569 (56.17%) |  |
| Living with partner | 58 (5.74%) | 59 (5.85%) | 47 (4.64%) |  |
| Never married | 109 (10.79%) | 96 (9.52%) | 133 (13.13%) |  |
| Other | 235 (23.27%) | 244 (24.21%) | 264 (26.06%) |  |
| PIR |  |  |  | 0.823 |
| High | 192 (19.01%) | 186 (18.45%) | 197 (19.45%) |  |
| Medium | 433 (42.87%) | 453 (44.94%) | 452 (44.62%) |  |
| Low | 385 (38.12%) | 369 (36.61%) | 364 (35.93%) |  |
| SMOKE |  |  |  | <0.001 |
| Never | 634 (62.77%) | 566 (56.15%) | 476 (46.99%) |  |
| Former | 252 (24.95%) | 284 (28.17%) | 332 (32.77%) |  |

Table S4A Continued

| Variables | Q1(n=1047) | Q2(n=1046) | Q3(n=1048) | *P*value |
| --- | --- | --- | --- | --- |
| Now | 124 (12.28%) | 158 (15.67%) | 205 (20.24%) |  |
| ALCOHOL USE |  |  |  | <0.001 |
| Never | 230 (22.77%) | 191 (18.95%) | 144 (14.22%) |  |
| Former | 167 (16.53%) | 149 (14.78%) | 157 (15.50%) |  |
| Mild | 324 (32.08%) | 379 (37.60%) | 414 (40.87%) |  |
| Moderate | 126 (12.48%) | 112 (11.11%) | 133 (13.13%) |  |
| Heavy | 163 (16.14%) | 177 (17.56%) | 165 (16.29%) |  |
| DIABETES |  |  |  | 0.008 |
| No | 782 (77.43%) | 759 (75.30%) | 724 (71.47%) |  |
| Yes | 228 (22.57%) | 249 (24.70%) | 289 (28.53%) |  |
| HYPERLIPIDEMIA |  |  |  | 0.025 |
| No | 700 (69.31%) | 711 (70.54%) | 755 (74.53%) |  |
| Yes | 310 (30.69%) | 297 (29.46%) | 258 (25.47%) |  |
| HYPERTENSION |  |  |  | <0.001 |
| No | 658 (65.15%) | 583 (57.84%) | 496 (48.96%) |  |
| Yes | 352 (34.85%) | 425 (42.16%) | 517 (51.04%) |  |
| PRECVD |  |  |  | <0.001 |
| No | 896 (88.71%) | 865 (85.81%) | 781 (77.10%) |  |
| Yes | 114 (11.29%) | 143 (14.19%) | 232 (22.90%) |  |

ALT: alanine aminotransferase; AST: aspartate aminotransferase; UACR: urine albumin to creatinine ratio; SIRI: systemic immune-inflammation index;
