# Supplementary material for: Association between the systemic inflammatory response index and mortality in patients with sarcopenia
Source: PLoS One. 2024 Nov 18;19(11):e0312383. doi: 10.1371/journal.pone.0312383 (PMC11573146; doi:10.1371/journal.pone.0312383)
Supplement: S4 Table — A. Characteristics of sarcopenia: excluding participants who died within two years in the NHANES study. B. Association of SIRI with all-cause and cause-specific mortality in sarcopenia participants (excluding participants who died within two years). (ZIP) [file pone.0312383.s007.zip › S4B_Table.docx]

Table S4B Association of SIRI with All-Cause and Cause-Specific Mortality in Sarcopenia Participants (Excluding Participants who Died within Two Years).

|  | All-cause mortality | | | | | |
| --- | --- | --- | --- | --- | --- | --- |
|  | Model 1 | | Model 2 | | Model 3 | |
| Character | 95%CI | *p* | 95%CI | *p* | 95%CI | *p* |
| Q1 | ref |  | ref |  | ref |  |
| Q2 | 1.50 (1.26, 1.78) | <0.0001 | 1.23 (1.04, 1.47) | 0.0189 | 1.16 (0.97, 1.39) | 0.0947 |
| Q3 | 2.06 (1.75, 2.43) | <0.0001 | 1.31 (1.11, 1.56) | 0.0018 | 1.19 (1.00, 1.42) | 0.0515 |
|  | Cardiovascular disease mortality | | | | | |
|  | Model 1 | | Model 2 | | Model 3 | |
| Character | 95%CI | *p* | 95%CI | *p* | 95%CI | *p* |
| Q1 | ref |  | ref |  | ref |  |
| Q2 | 1.73 (1.22, 2.45) | 0.0020 | 1.37 (0.96, 1.95) | 0.0821 | 1.32 (0.92, 1.90) | 0.1295 |
| Q3 | 2.48 (1.78, 3.45) | <0.0001 | 1.48 (1.05, 2.09) | 0.0267 | 1.37 (0.96, 1.95) | 0.0848 |
|  | Cancer Diseases mortality | | | | | |
|  | Model 1 | | Model 2 | | Model 3 | |
| Character | 95%CI | *p* | 95%CI | *p* | 95%CI | *p* |
| Q1 | ref |  | ref |  | ref |  |
| Q2 | 1.13 (0.78, 1.63) | 0.5085 | 0.91 (0.62, 1.32) | 0.6083 | 0.87 (0.59, 1.27) | 0.4581 |
| Q3 | 1.49 (1.05, 2.12) | 0.0244 | 0.94 (0.65, 1.36) | 0.7274 | 0.85 (0.58, 1.25) | 0.4141 |
|  | Respiratory diseases mortality | | | | | |
|  | Model 1 | | Model 2 | | Model 3 | |
| Character | 95%CI | *p* | 95%CI | *p* | 95%CI | *p* |
| Q1 | ref |  | ref |  | ref |  |
| Q2 | 1.76 (0.77, 4.03) | 0.1792 | 1.28 (0.55, 2.96) | 0.5709 | 1.10 (0.47, 2.59) | 0.8247 |
| Q3 | 3.29 (1.54, 7.01) | 0.0021 | 1.75 (0.79, 3.86) | 0.1680 | 1.51 (0.68, 3.39) | 0.3145 |

Model 1: No adjustment for covariates. Model 2: adjusted for age, gender, and race. Model 3: Age, gender, race, education, household income to poverty ratio, marital status, smoking status, drinking status, diabetes, hypertension, hyperlipidemia, UACR, ALT, and AST.
