# Supplementary material for: Association between the systemic inflammatory response index and mortality in patients with sarcopenia
Source: PLoS One. 2024 Nov 18;19(11):e0312383. doi: 10.1371/journal.pone.0312383 (PMC11573146; doi:10.1371/journal.pone.0312383)
Supplement: S5 Table — A. Characteristics of sarcopenia participants in the NHANES (excluding participants under the age of 50). B. Association of SIRI with all-Cause and cause-specific mortality in sarcopenia participants(excluding participants under the age of 50). (ZIP) [file pone.0312383.s008.zip › S5A_Table.docx]

Table S5A Characteristics of Sarcopenia Participants in the NHANES (Excluding Participants under the age of 50).

| Variables | Q1(n=681) | Q2(n=683) | Q3(n=683) | *P*value |
| --- | --- | --- | --- | --- |
| AGE | 63.42 ± 9.46 | 66.80 ± 10.71 | 69.25 ± 10.72 | <0.001 |
| ALT | 25.85 ± 13.86 | 24.06 ± 13.50 | 24.05 ± 13.16 | 0.018 |
| AST | 26.40 ± 12.36 | 24.71 ± 9.87 | 24.68 ± 9.95 | 0.003 |
| UACR | 88.73 ± 744.31 | 116.13 ± 670.02 | 99.07 ± 332.88 | 0.703 |
| SIRI | 0.67 ± 0.16 | 1.17 ± 0.16 | 2.37 ± 1.21 | <0.001 |
| GENDER |  |  |  | <0.001 |
| Male | 264 (38.77%) | 348 (50.95%) | 438 (64.13%) |  |
| Female | 417 (61.23%) | 335 (49.05%) | 245 (35.87%) |  |
| RACE |  |  |  | <0.001 |
| Mexican American | 319 (46.84%) | 255 (37.34%) | 173 (25.33%) |  |
| Other Hispanic | 51 (7.49%) | 52 (7.61%) | 35 (5.12%) |  |
| Non-Hispanic White | 196 (28.78%) | 319 (46.71%) | 417 (61.05%) |  |
| Non-Hispanic Black | 48 (7.05%) | 24 (3.51%) | 35 (5.12%) |  |
| Other Race Including Multi-Racial | 67 (9.84%) | 33 (4.83%) | 23 (3.37%) |  |
| EDUCATION |  |  |  | 0.001 |
| Less Than 9th Grade | 229 (33.63%) | 244 (35.72%) | 186 (27.23%) |  |
| 9-11th Grade (Includes 12th grade with no diploma) | 119 (17.47%) | 88 (12.88%) | 108 (15.81%) |  |
| High School Grad/GED or Equivalent | 149 (21.88%) | 139 (20.35%) | 160 (23.43%) |  |
| Some College or AA degree | 104 (15.27%) | 146 (21.38%) | 140 (20.50%) |  |
| College Graduate or above | 80 (11.75%) | 66 (9.66%) | 89 (13.03%) |  |
| MARITAL STATUS |  |  |  | 0.272 |
| Married | 414 (60.79%) | 424 (62.08%) | 395 (57.83%) |  |
| Living with partner | 13 (1.91%) | 19 (2.78%) | 18 (2.64%) |  |
| Never married | 43 (6.31%) | 27 (3.95%) | 35 (5.12%) |  |
| Other | 211 (30.98%) | 213 (31.19%) | 235 (34.41%) |  |
| PIR |  |  |  | 0.922 |
| High | 138 (20.26%) | 136 (19.91%) | 129 (18.89%) |  |
| Medium | 311 (45.67%) | 313 (45.83%) | 327 (47.88%) |  |
| Low | 232 (34.07%) | 234 (34.26%) | 227 (33.24%) |  |
| SMOKE |  |  |  | <0.001 |
| Never | 395 (58.00%) | 339 (49.63%) | 278 (40.70%) |  |

Table S5A Continued

| Variables | Q1(n=681) | Q2(n=683) | Q3(n=683) | *P*value |
| --- | --- | --- | --- | --- |
| Former | 211 (30.98%) | 254 (37.19%) | 285 (41.73%) |  |
| Now | 75 (11.01%) | 90 (13.18%) | 120 (17.57%) |  |
| ALCOHOL USE |  |  |  | <0.001 |
| Never | 160 (23.49%) | 128 (18.74%) | 101 (14.79%) |  |
| Former | 130 (19.09%) | 135 (19.77%) | 124 (18.16%) |  |
| Mild | 238 (34.95%) | 301 (44.07%) | 317 (46.41%) |  |
| Moderate | 79 (11.60%) | 55 (8.05%) | 66 (9.66%) |  |
| Heavy | 74 (10.87%) | 64 (9.37%) | 75 (10.98%) |  |
| DIABETES |  |  |  | 0.188 |
| No | 481 (70.63%) | 465 (68.08%) | 451 (66.03%) |  |
| Yes | 200 (29.37%) | 218 (31.92%) | 232 (33.97%) |  |
| HYPERLIPIDEMIA |  |  |  | 0.126 |
| No | 455 (66.81%) | 455 (66.62%) | 486 (71.16%) |  |
| Yes | 226 (33.19%) | 228 (33.38%) | 197 (28.84%) |  |
| HYPERTENSION |  |  |  | <0.001 |
| No | 365 (53.60%) | 276 (40.41%) | 238 (34.85%) |  |
| Yes | 316 (46.40%) | 407 (59.59%) | 445 (65.15%) |  |
| PRECVD |  |  |  | <0.001 |
| No | 138 (20.26%) | 136 (19.91%) | 129 (18.89%) |  |
| Yes | 138 (20.26%) | 136 (19.91%) | 129 (18.89%) |  |
| Former | 211 (30.98%) | 254 (37.19%) | 285 (41.73%) |  |
| Now | 75 (11.01%) | 90 (13.18%) | 120 (17.57%) |  |
| ALCOHOL USE |  |  |  | <0.001 |
| Never | 160 (23.49%) | 128 (18.74%) | 101 (14.79%) |  |
| Former | 130 (19.09%) | 135 (19.77%) | 124 (18.16%) |  |
| Mild | 238 (34.95%) | 301 (44.07%) | 317 (46.41%) |  |
| Moderate | 79 (11.60%) | 55 (8.05%) | 66 (9.66%) |  |
| Heavy | 74 (10.87%) | 64 (9.37%) | 75 (10.98%) |  |
| DIABETES |  |  |  | 0.188 |
| No | 481 (70.63%) | 465 (68.08%) | 451 (66.03%) |  |
| Yes | 200 (29.37%) | 218 (31.92%) | 232 (33.97%) |  |

ALT: alanine aminotransferase; AST: aspartate aminotransferase; UACR: urine albumin to creatinine ratio; SIRI: systemic immune-inflammation index;
