# Supplementary material for: Association between the systemic inflammatory response index and mortality in patients with sarcopenia
Source: PLoS One. 2024 Nov 18;19(11):e0312383. doi: 10.1371/journal.pone.0312383 (PMC11573146; doi:10.1371/journal.pone.0312383)
Supplement: S5 Table — A. Characteristics of sarcopenia participants in the NHANES (excluding participants under the age of 50). B. Association of SIRI with all-Cause and cause-specific mortality in sarcopenia participants(excluding participants under the age of 50). (ZIP) [file pone.0312383.s008.zip › S5B_Table.docx]

Table S5B Association of SIRI with All-Cause and Cause-Specific Mortality in Sarcopenia Participants(Excluding Participants under the age of 50).

|  | All-cause mortality | | | | | |
| --- | --- | --- | --- | --- | --- | --- |
|  | Model 1 | | Model 2 | | Model 3 | |
| Character | 95%CI | *p* | 95%CI | *p* | 95%CI | *p* |
| Q1 | ref |  | ref |  | ref |  |
| Q2 | 1.65 (1.40, 1.94) | <0.0001 | 1.28 (1.08, 1.51) | 0.0038 | 1.22 (1.03, 1.45) | 0.0221 |
| Q3 | 2.16 (1.84, 2.54) | <0.0001 | 1.36 (1.15, 1.61) | 0.0003 | 1.26 (1.06, 1.50) | 0.0080 |
|  | Cardiovascular disease mortality | | | | | |
|  | Model 1 | | Model 2 | | Model 3 | |
| Character | 95%CI | *p* | 95%CI | *p* | 95%CI | *p* |
| Q1 | ref |  | ref |  | ref |  |
| Q2 | 1.72 (1.24, 2.40) | 0.0012 | 1.27 (0.91, 1.78) | 0.1589 | 1.20 (0.86, 1.69) | 0.2869 |
| Q3 | 2.60 (1.90, 3.56) | <0.0001 | 1.52 (1.09, 2.11) | 0.0124 | 1.37 (0.98, 1.91) | 0.0651 |
|  | Cancer Diseases mortality | | | | | |
|  | Model 1 | | Model 2 | | Model 3 | |
| Character | 95%CI | *p* | 95%CI | *p* | 95%CI | *p* |
| Q1 | ref |  | ref |  | ref |  |
| Q2 | 1.21 (0.86, 1.72) | 0.2754 | 0.86 (0.57, 1.28) | 0.4524 | 0.84 (0.56, 1.26) | 0.3970 |
| Q3 | 1.64 (1.17, 2.29) | 0.0038 | 1.01 (0.71, 1.45) | 0.9399 | 0.97 (0.67, 1.39) | 0.8558 |
|  | Respiratory diseases mortality | | | | | |
|  | Model 1 | | Model 2 | | Model 3 | |
| Character | 95%CI | *p* | 95%CI | *p* | 95%CI | *p* |
| Q1 | ref |  | ref |  | ref |  |
| Q2 | 1.33 (0.59, 2.96) | 0.4915 | 0.97 (0.43, 2.19) | 0.9435 | 1.02 (0.45, 2.31) | 0.9675 |
| Q3 | 3.23 (1.61, 6.49) | 0.0010 | 1.82 (0.87, 3.80) | 0.1111 | 1.76 (0.84, 3.69) | 0.1352 |

Model 1: No adjustment for covariates. Model 2: adjusted for age, gender, and race. Model 3: Age, gender, race, education, household income to poverty ratio, marital status, smoking status, drinking status, diabetes, hypertension, hyperlipidemia, UACR, ALT, and AST.
